# Incidental and Clinical Significance of Slit Ventricles in Fixed Pressure Valves

**DOI:** 10.7759/cureus.30902

**Published:** 2022-10-31

**Authors:** Khalid T Alghamdi, Mohammed D Alghamdi, Sultan Neazy, Mohannd M Algamdi, Abdullah Alzahrani, Muhammad A Khan, Abdulhadi Algahtani

**Affiliations:** 1 College of Medicine, King Saud Bin Abdulaziz University for Health Sciences, King Abdullah International Medical Research Centre, Jeddah, SAU; 2 Internal Medicine, King Saud Bin Abdulaziz University for Health Sciences, King Abdullah International Medical Research Centre, Jeddah, SAU; 3 College of Medicine, King Saud Bin Abdulaziz University for Health Sciences, Jeddah, SAU; 4 General Surgery, King Saud Bin Abdulaziz University for Health Sciences, King Abdullah International Medical Research Centre, Jeddah, SAU; 5 Epidemiology and Public Health, King Saud Bin Abdulaziz University, Jeddah, SAU; 6 Neurological Surgery, King Saud Bin Abdulaziz University for Health Sciences College of Medicine, Jeddah, SAU

**Keywords:** hydrocephalus, hydrocephalus management, fixed pressure valve, slit-like ventricle, slit ventricular syndrome (svs)

## Abstract

Introduction: Slit ventricle syndrome (SVS) is a recognized delayed complication of cerebrospinal fluid (CSF) shunting in children. It had been linked to the use of low-pressure shunts and considered an argument for the use of programmable valves. In this study, we aim to assess the rate of SVS in children that were shunted using fixed-pressure valves.

Methodology: This study is a retrospective cohort study that occurred in King Abdulaziz Medical City, Jeddah, which reviews 100 patients with a median age of 15.5 months that were shunted by using fixed pressure valves during the period from 2010 to 2018. Fixed low-pressure valves were used in 69% of patients, while fixed medium-pressure valves were used in 31% of patients. SVS was defined by the presence of slit-like ventricles (fronto-occipital [F-O] horns ratio was ≤ 0.2 on any post-shunt CT scan) and the occurrence of slit-like ventricle-related symptoms (chronic headache, nausea, vomiting, and altered conscious level_ in the absence of other causes of shunt malfunction.

Results: The overall SVS rate in the cohort was 6%. Nine children had slit-like ventricles, but only six of them were symptomatic. Relatively higher SVS rates were observed in younger male children, obstructive hydrocephalus, and medium-pressure valves. Slit-like ventricle-related symptoms in the absence of a slit-like ventricle were reported in 24 out of 91 (26%) patients. A total of 42 patients underwent shunt revisions for other complications. All SVS patients were treated conservatively. There was a temporal fluctuation in the F-O horns ratio and in some patients with SVS their F-O horns ratio returned to normal at further follow-up without intervention.

Conclusions: The overall SVS rate following the use of fixed-pressure CSF valves in children is low and managed conservatively. Not all patients with slit-like ventricles are symptomatic and the radiological appearance of SVS may improve on further follow-up without intervention. Fixed pressure valves remain an acceptable device in the treatment of hydrocephalus in children.

## Introduction

Hydrocephalus is known as the active accumulation of the cerebrospinal fluid (CSF) leading to an increase in the space of the brain’s ventricular system and the pressure on the surrounding structures [1]. The prevalence rate accounts for six out of 10,000 patients in the pediatric age group. There are three main types of hydrocephalus: communicating, obstructive (non-communicating), and ex vacuo hydrocephalus, which usually affect elderly patients [2]. The time and duration of hydrocephalus may largely affect the brain development process, especially in the pediatric age group [3]. The treatment plan should be individualized for each patient. Moreover, it consists mainly of endoscopic procedures and shunt implantation [4]. There are two types of valves that are used to treat patients with hydrocephalus which are fixed and adjustable pressure valves. Fixed pressure (non-programmable valve) is a valve that controls the CSF flow with an already set differential pressure that cannot be readjusted after an invasive procedure. Fixed pressure valves commonly come in five ranges which are: very low, low, medium, high, and very high pressures. An adjustable pressure valve (programmable valve) is a valve that can be adjusted non-invasively to change its differential pressure [5]. It has an external adjustment tool that can select a specific differential pressure setting from a range of different settings [6]. Complications of a shunt might happen such as over or under-drainage of CSF [5]. One of the complications of hydrocephalus patients with a shunt is slit ventricle syndrome (SVS). SVS is a combination of a decrease in fronto-occipital (F-O) horns ratio on the brain computed tomography (CT) scan with intracranial pressure-related symptoms like vomiting, severe intermittent headache, and loss of consciousness [7].

This study aims to evaluate the incidence and clinical significance of SVS in pediatric patients who have fixed pressure valves regardless of the hydrocephalus type in King Abdulaziz Medical City in Jeddah from January 2010 to December 2018 to enhance the management and outcome of pediatric patients with hydrocephalus who undergo shunting procedure.

## Materials and methods

This is a retrospective observational study of 100 patients that had hydrocephalus and underwent a ventriculoperitoneal (VP) shunting procedure using fixed pressure valves at King Abdulaziz Medical City (KAMC), Jeddah from January 2010 to December 2018. The study includes patients aged ≤ 16 years, that had a minimal follow-up duration of two years and had adequate post-shunting clinical and imaging evaluations.

The definition of slit-like ventricles was made by measuring the F-O horns ratio on the brain CT scan. It was calculated by dividing the mean of the bifrontal and bioccipital horns distances over the maximum cranial interparietal distance. The F-O horns ratio is normally around 0.37 and hence, a ratio of ≤ 0.2 at any time, was considered diagnostic of a slit-like ventricle [7]. The definition of SVS was based on the reporting of clinical symptoms such as chronic headache, nausea, vomiting, and altered conscious state, in the presence of slit-like ventricles and the absence of other identifiable causes of shunt malfunction by imaging [8]. The definition of SVS was based on the presence of slit-like ventricles and slit-like ventricle-related symptoms [8].

Data collection method

Data collection was done by collecting the patients’ hospital records and KAMC’s Information System, BESTCare system. The data included the patient’s age at presentation, type of hydrocephalus, type of fixed pressure valve, number, causes and timing of all shunt revisions, the presence of slit-like ventricle-related symptoms, timing of their occurrence, and how they were managed.

F-O horns ratio measurement was done at the presentation and all the available follow-up points for all the patients. Patients who had at least one F-O horns ratio ≤ 0.2 in their post-shunting follow-up evaluations were referred to as the slit-like ventricles group. The remaining patients were referred to as the non-slit-like ventricles group. The two groups were compared based on age, gender, type of fixed pressure valve used, number and cause of shunt revisions, and the reporting of slit-like ventricle-related symptoms. The temporal changes in the F-O horns ratio were compared between the two groups.

Statistical presentation

The demographic data and the clinical characteristics were presented as frequency and percentages (categorical variables), while numerical variables were shown as median and range.

Ethical approval 

The study was approved by the institutional review board (IRB) of King Abdullah International Medical Research Center (KAIMRC) with the number NRJ21J/049/02 on May 4, 2021.

## Results

Demographics

The study included 100 patients who underwent ventriculoperitoneal shunt with fixed pressure valves from January 2010 to December 2018. The median follow-up duration was 6 years. Out of these, 50 patients were males, and 50 were females. The median age was 15.5 months (0.03- 180). Most patients were diagnosed with obstructive hydrocephalus, which accounts for 70 patients, 14 patients were having to communicate hydrocephalus, and for the remaining 16 patients, the hydrocephalus type was not specified. In addition to that, 69 patients were using fixed low-pressure valves, and 31 were using fixed moderate-pressure valves. In contrast, a fixed high-pressure valve was not used to treat patients in our study.

Follow-up median comparison

**Figure 1:**
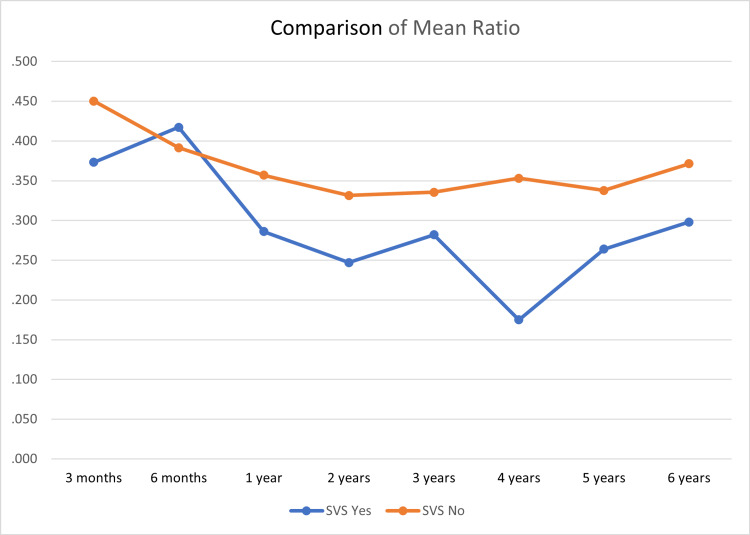
The mean frontal and occipital ratios of patients during their follow-up SVS = Slit Ventricle Syndrome

Of 100 patients, six patients (6%) presented with SVS. Nine children had slit-like ventricles, but only six of them were symptomatic. However, the frontal and occipital ratios were fluctuating. In some of the patients who developed SVS, the ratio was resolved to the normal level after the development of SVS. The median (range) F-O horns ratio for the 100 patients was 0.356 (0.098- 0.88) (Figure 1).

Clinical characteristics comparison

The incidence of SVS in males was higher than in females (2:1 ratio). The types of Medtronic Parkway Minneapolis Medical fixed pressure valves were used on our patients as the following. Sixty-nine (69%) patients were given low pressure valve, and 31 (31%) patients were given medium pressure valve. No high-pressure valves were used to treat patients in our study. Shunt revisions were done in a total number of 42 patients. Twenty-four patients had one revision, 14 patients had two revisions, two patients had three revisions, and two patients had four revisions. In the patients who underwent at least one revision, the median of shunt revision was 1. The cause of shunt revisions was an infection in 18 revisions (nine patients) and shunt malfunction in 42 revisions (33 patients). The revisions were with the same model of shunt. These patients reported many symptoms such as headache, nausea/vomiting, sleepiness/decreased level of consciousness, and seizure. In addition to that, there was no significant difference in the reporting of symptoms among them. Indeed, some of the symptoms were found in patients without SVS such as sleepiness/decreased level of consciousness (Table 1). However, headache and nausea/vomiting were reported in patients with SVS more frequently (Table 2). Regarding the management, all the patients were managed conservatively as their symptoms were mild and their frontal and occipital ratios retained to the normal level spontaneously.

**Table 1:** Clinical characteristics of the patients in the study

		Slit-like ventricle			
		No		Yes	
		n	%	n	%
Age	≤Median	44	88.0	6	12.0
	>Median	47	94.0	3	6.0
Gender	Male	44	88.0	6	12.0
	Female	47	94.0	3	6.0
The type of hydrocephalus	Communicated	13	92.9	1	7.1
	Obstructed	63	90.0	7	10.0
	Unspecified	15	93.8	1	6.2
The type of fixed pressure valves	Low	65	94.2	4	5.8
	Intermediate	26	83.9	5	16.1
Revision	≤ 1	73	93.6	5	6.4
	> 1	18	81.8	4	18.2
Cause of revision	Malfunction	29	87.9	4	12.1
	Infection	7	88.9	2	22.2
Symptomatic	Symptomatic	24	80	6	20
	Asymptomatic	67	95.7	3	4.3
Headache	Yes	14	77.8	4	22.2
	No	77	93.9	5	6.1
Nausea/Vomiting	Yes	15	78.9	4	21.1
	No	76	93.6	5	6.2
Sleepiness/Decrease level of consciousness	Yes	7	100.0	0	0.0
	No	84	90.3	9	9.7

**Table 2:** Clinical characteristics of slit-like ventricle cases in the study

SV cases	Age (months)	Gender	Type of hydrocephalus	Type of fixed-pressure valve	Number of Revisions	Cause of revision	Symptoms
1	84	Male	Obstructive	Low	2	Shunt malfunction(obstruction)	Headache
2	2	Male	Unspecified	Intermediate	1	Shunt malfunction(obstruction)	Nausea/vomiting
3	108	Male	Obstructive	Intermediate	0	-	Headache, Nausea/vomiting
4	3	Male	Communicating	Low	2	Shunt malfunction(obstruction)	Nausea/vomiting
5	1	Female	Obstructive	Intermediate	3	Shunt malfunction(obstruction), Infection	Headache
6	120	Female	Obstructive	Intermediate	0	-	Headache, nausea/vomiting
7	4	Male	Obstructive	Low	2	Infection	Asymptomatic
8	0.03	Female	Obstructive	Intermediate	0	-	Asymptomatic
9	1.8	Male	Obstructive	Low	0	-	Asymptomatic

## Discussion

SVS is a complication of hydrocephalus resulting in the appearance of a “slit-like” ventricle in the CT scan or magnetic resonance imaging (MRI) [8]. The criteria for SVS diagnosis include having an F-O ratio of 0.2 on the brain CT scan in addition to the appearance of slit-like ventricle-related symptoms such as intermittent headache [7]. The choices of valves for treating a patient with hydrocephalus are divided mainly into fixed and adjustable pressure valves. Regarding the fixed pressure valve, the set differential pressure cannot be changed and is available in different scales as follows: very low, low, medium, high, and very high pressure. On the other hand, the pressure range can be changed in the adjustable valves without the need for valve replacement by invasive interventions [5]. There is a large difference in the costs of the two valves as per published articles. For instance, a study conducted in Pennsylvania revealed that the cost of the adjustable valve was $3,438 (interquartile range $2,938-$3,876) and the fixed valve was $1,504 (interquartile range $753-$1,584). Moreover, there was no major difference in the morbidity and mortality rates between the two different valves [9]. Thus, there is a need to consider the cost-effectiveness when using the adjustable instead of the fixed pressure valve.

In one study that involved 244 patients who were treated with adjustable pressure valves, 23 (9.4%) of them had a radiological finding of slit-like ventricles, and from those, two required shunt revision [10]. In other study that included 370 patients treated with adjustable pressure valves, 64% of them showed to have slit-like ventricles radiologically. Furthermore, a small percentage of patients developed SVS (11.5%). 6.5% of the patients who had SVS required treatment, but the other 5% did not require any sort of management [11]. In our study, from 100 patients who were treated with fixed pressure valves, only six (6%) of patients developed SVS. Also, all SVS patients did not require any intervention, and their ratios normalized spontaneously. Furthermore, regarding the relation between the valve type and the incidence of SVS, we found that there was not any big difference between fixed or adjustable pressure valves.

Slit ventricle could result from shunt over-drainage or shunt malfunction. one of the complications of slit ventricle is shunt over-drainage, so it is important to understand and discuss the mechanism of shunt over-drainage because itself is considered a well-known complication in hydrocephalus management. The exact mechanism of shunt over-drainage is not clearly understood, but multiple theories describe the pathophysiology behind it. The first one leads to intracranial hypotension due to over-drainage that is related to siphoning, and the remaining theories cause intracranial hypertension due to over-drainage related to slow vasogenic waves. The first theory is siphoning theory, which explains the role of postural change by the law of Steven: PP = (ICP - IAP) + HP, where PP is the perfusion pressure, ICP the intracranial pressure, IAP the intra-abdominal, and HP is the hydrostatic pressure. A ventricular emptying with intracranial hypotension occurs when the patient goes from decubitus to an upright position due to gravity between the ventricles and the peritoneal cavity. The second theory is ventricular collapse and cerebrospinal fluid isolation, which result from distortion of cerebral structures with CSF isolation in ventricular compartments or the subarachnoid space. The third theory is acquired craniocerebral disproportion. This theory occurs in newborns and infant patients when CSF shunting leads to early suture ossification with skull remodeling. This will lead to an impairment in intracranial compliance with intracranial hypertension and sometimes cerebral tonsillar herniation (acquired Chiari). The last theory is venous hypertension theory, which results from diffuse meningeal venous hyperemia and engorgement of venous sinuses. Hence, it increases elasticity and stiffness as this depends mainly on the functioning of the cerebral venous outflow mechanism [12].

There were some contrasting and similar points between our study and other studies. Starting with gender differences, the male-to-female ratio was 2:1, with six male patients and three female patients. In another study that was done in 1985 on four patients with SVS, the male-to-female ratio was 1:1, two males and two females. This could raise a question of whether male sex is a risk factor or not. Regarding revisions, five SVS patients required one or less revisions, and four of them needed more than one revision. While in the other study, all four patients needed revisions several times. This could be due to the advancement in practice [13]. In our study, six of the nine patients with slit-like ventricles were symptomatic. For example, a headache was reported in four patients, nausea/vomiting was reported in four patients, and none of them had sleeplessness/decreased level of consciousness. On the other hand, in the other research, all the 16 patients who had SVS had the intracranial pressure-related symptoms which were mentioned previously [14]. Furthermore, in a study that included 164 patients, eight of them had SVS. Headache and vomiting were the prominent symptoms, which one or both of them occurred in all patients. Sleeplessness/decreased level of consciousness occurred in three patients. One patient had somnolence, one had stupor, and one had coma. In summary, among symptoms, headache and vomiting were the most common symptoms to occur while Sleeplessness/decreased level of consciousness was not found in our research as opposed to the other study [15]. This study was limited by being in a single center. Also, the file system, where some of the data were collected, was a limitation as some of the information was not found in the files.

## Conclusions

Allowing for the increase in cost with the use of programmable valves, our findings suggest that fixed-pressure valves remain at the present time an acceptable device in the treatment of hydrocephalus in children. The SVS incidence following the use of fixed pressure valves in children is small and usually can be managed conservatively. Not all patients with slit-like ventricles are symptomatic and the radiological appearance of slit-like ventricles may improve on further follow-up without intervention. Headache and nausea/vomiting are the prominent symptoms in patients with SVS while sleeplessness/decreased level of consciousness does not appear to be prominent. Since there is a lack of studies in our region, we encourage conducting further research that involves multiple centers.
